# Targeting AURKA in Cancer: molecular mechanisms and opportunities for Cancer therapy

**DOI:** 10.1186/s12943-020-01305-3

**Published:** 2021-01-15

**Authors:** Ruijuan Du, Chuntian Huang, Kangdong Liu, Xiang Li, Zigang Dong

**Affiliations:** 1 Department of Pathophysiology, School of Basic Medical Sciences, Zhengzhou University, Zhengzhou, 450001 Henan China; 2 China-US (Henan) Hormel Cancer Institute, No. 127, Dongming Road, Jinshui District, Zhengzhou, 450008 Henan China; 3 The Collaborative Innovation Center of Henan Province for Cancer Chemoprevention, Zhengzhou, China; 4 State Key Laboratory of Esophageal Cancer Prevention and Treatment, Zhengzhou University, Zhengzhou, Henan China; 5 College of medicine, Zhengzhou University, Zhengzhou, 450001 Henan China

**Keywords:** Aurora kinase a, Cancer, Regulators, Substrates, Inhibitors, Combination therapy

## Abstract

Aurora kinase A (AURKA) belongs to the family of serine/threonine kinases, whose activation is necessary for cell division processes via regulation of mitosis. AURKA shows significantly higher expression in cancer tissues than in normal control tissues for multiple tumor types according to the TCGA database. Activation of AURKA has been demonstrated to play an important role in a wide range of cancers, and numerous AURKA substrates have been identified. AURKA-mediated phosphorylation can regulate the functions of AURKA substrates, some of which are mitosis regulators, tumor suppressors or oncogenes. In addition, enrichment of AURKA-interacting proteins with KEGG pathway and GO analysis have demonstrated that these proteins are involved in classic oncogenic pathways. All of this evidence favors the idea of AURKA as a target for cancer therapy, and some small molecules targeting AURKA have been discovered. These AURKA inhibitors (AKIs) have been tested in preclinical studies, and some of them have been subjected to clinical trials as monotherapies or in combination with classic chemotherapy or other targeted therapies.

## Introduction

Aurora kinases belong to serine/threonine kinases which share a highly conserved catalytic domain containing auto-phosphorylating sites. This family contains three members: Aurora A (AURKA), Aurora B (AURKB), and Aurora C (AURKC). Both AURKA and AURKB play essential roles in regulating cell division during mitosis while AURKC has a unique physiological role in spermatogenesis. Relatively less information is available for the roles of AURKC in cancer. AURKA and AURKB have been found to function as oncogenes to promote tumorigenesis in multiple types of cancer including solid tumors and hematological malignancies. Even though, AURKA has attracted researchers’ attentions and has been a more popular target than AURKB for cancer therapy with nearly fifty clinical trials using specific AKIs. However, only about ten clinical trials using inhibitors specifically targeting AURKB and most of them are still in phase I stage. In comparison, the most popular AKI alisertib has finished phase III clinical assessment. In this review, we will focus on research progress associated with AURKA in cancer. Apart from playing a role in mitosis, an increasing number of studies have suggested that AURKA, when abnormally expressed, could be an oncogene involved in tumorigenesis. Gene amplification, transcriptional activation and inhibition of protein degradation could contribute to the elevated levels of AURKA expression in cancer tissues. AURKA promotes tumorigenesis by participating in the cancer cell proliferation, epithelial-mesenchymal transition (EMT), metastasis, apoptosis, and self-renewal of cancer stem cells. Given that overexpression and gene amplification of AURKA have been identified in diverse cancers, small molecule kinase inhibitors of AURKA have attracted considerable interest. A series of AURKA kinase inhibitors (AKIs) have been produced over the past decades; inhibition of the expression or activity of AURKA by AKIs suppresses cancer cell proliferation, migration and invasion. Excitingly, some AKIs have already been used in clinical trials. In this review, we highlight the importance of AURKA in cancer cell signal transduction. Moreover, we provide a summary of the selective inhibitors and pan-inhibitors of AURKA tested in various preclinical and clinical studies for the treatment of cancer.

### Expression of Aurora kinases in cancer

**Fig. 1:**
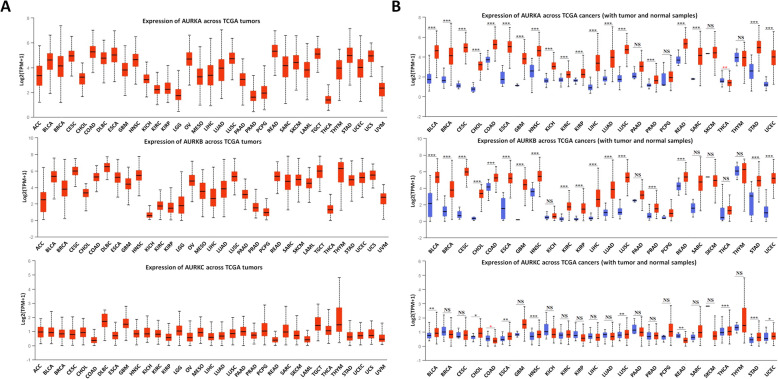
Expression of Aurora kinases in cancer. (A) Expression of Aurora kinases among various cancer types. (B) Comparison of the expression of Aurora kinases between tumor and normal tissues. The images and significance are from ULCAN database. * *P* < 0.05, ** *P* < 0.01, *** *P* < 0.001, NS: no significance

Aurora kinases are expressed in a wide range of cancers according to The Cancer Genome Atlas (TCGA) UALCAN database. As shown in Fig. 1a, AURKA expression is lowest in the thyroid carcinoma (THCA) dataset (median value 1.384) and highest in the rectum adenocarcinoma (READ) dataset (median value 5.329). AURKB has the lowest expression in the kidney chromophobe carcinoma (KICH) dataset (median value 0.576) and the highest expression in the diffuse large B-cell lymphoma (DLBC) dataset (median value 6.525). Four out of 33 (12.1%) cancer types show expression of AURKA with log2 (transcripts per million [TPM] + 1) values < 2, including brain lower-grade glioma (LGG), prostate adenocarcinoma (PRAD), pheochromocytoma and paraganglioma (PCPG) and THCA versus other tumors. In contrast, as many as 7 out of 33 (21.2%) cancer types show AURKB expression with a log2 (TPM + 1) value < 2. Moreover, all cancers exhibit AURKC expression with a log2 (TPM + 1) value < 2.

As shown in Fig. 1b**,** compared with normal tissues, most tumor types show significantly higher expression of AURKA, except for pancreatic adenocarcinoma (PAAD), PCPG, skin cutaneous melanoma (SKCM), and thymoma (THYM). Notably, AURKA expression is reduced in tumor tissues versus normal tissues in the THCA dataset. In samples from patients with 27 out of 33 tumor types, excluding KICH, PAAD, sarcoma (SARC), SKCM, THCA and THYM, AURKB has markedly higher expression in tumor tissues than in normal tissues. In contrast, AURKC expression is higher in tumor tissues than in normal tissues only in samples from patients with 9 out of 33 tumor types, including bladder urothelial carcinoma (BLCA), cholangiocarcinoma (CHOL), esophageal carcinoma (ESCA), head and neck squamous cell carcinoma (HNSC), lung squamous cell carcinoma (LUSC), READ, THCA and stomach adenocarcinoma (STAD). These data suggest that AURKA and AURKB are better potential targets than AURKC for cancer treatment.

### Significance of Aurora kinases expression

**Fig. 2:**
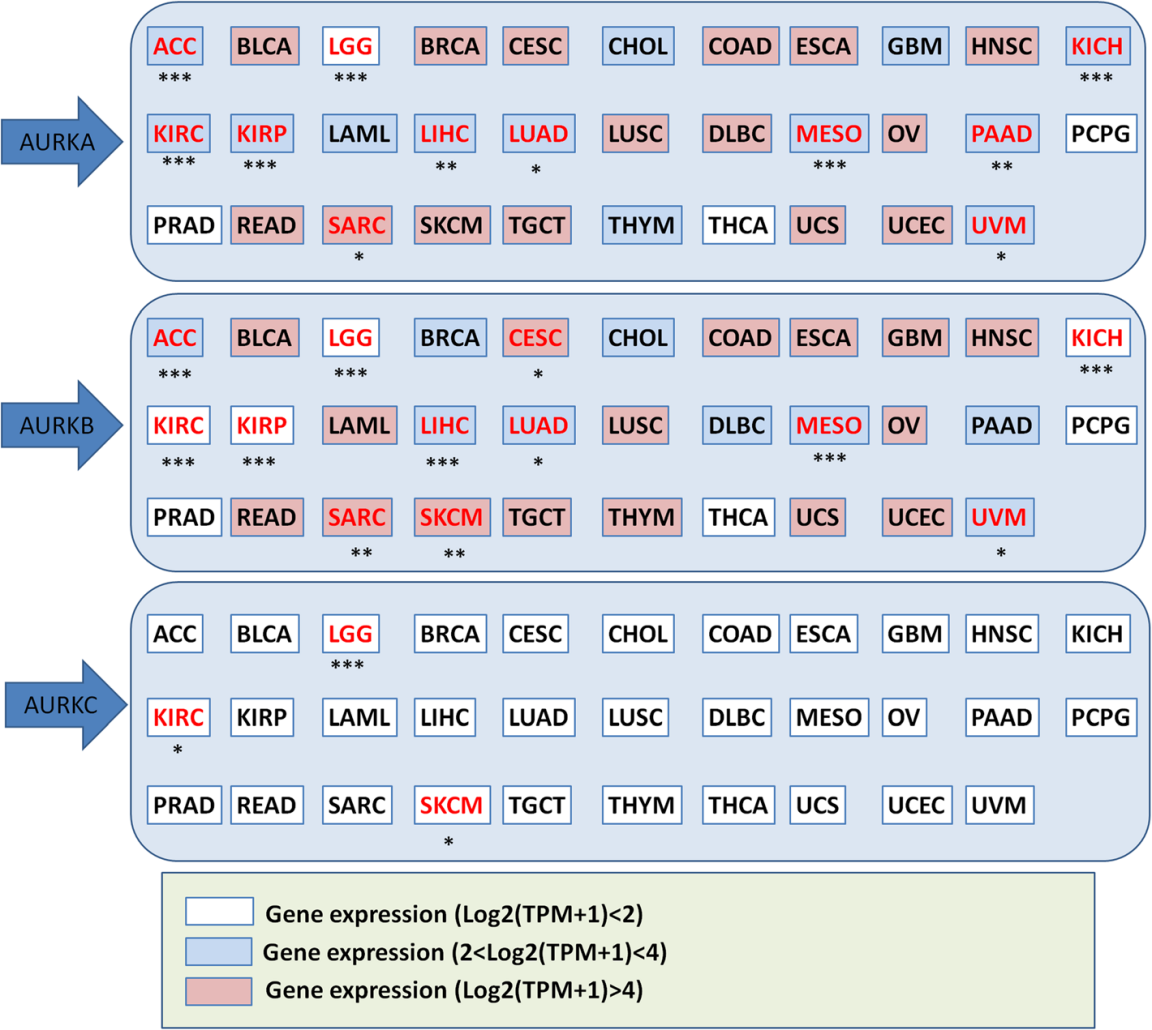
Correlation between Aurora kinases expression and patient overall survival. Red text: gene expression had significant relation with survival; black text: gene expression had no significant relation with survival. The survival data are derived from ULCAN database. Samples were categorized into two groups for analysis: High AURKA expression (with TPM values above upper quartile); Low/Medium AURKA expression (with TPM values below upper quartile). * *P* < 0.05, ** *P* < 0.01, *** *P* < 0.001

According to the TCGA UALCAN database, high expression of AURKA may be a sensitive prognostic marker in adrenocortical carcinoma (ACC), LGG, KICH, kidney renal clear cell carcinoma (KIRC), kidney renal papillary cell carcinoma (KIRP), liver hepatocellular carcinoma (LIHC), lung adenocarcinoma (LUAD), mesothelioma (MESO), PAAD, SARC and uveal melanoma (UVM). High AURKB expression was more closely related to worse overall survival in ACC, LGG, cervical squamous cell carcinoma (CESC), KICH, KIRC, KIRP, LIHC, LUAD, MESO, SARC, SKCM and UVM. Interestingly, AURKA and AURKB show similar patterns of survival correlation in ACC, LGG, KICH, KIRC, KIRP, LIHC, LUAD, MESO, SARC and UVM. Targeting both AURKA and AURKB in tumors of these cancer types may exert considerable antitumor effects. However, the expression of AURKC can predict patient survival only in LGG, KIRC and SKCM. These survival data are summarized in Fig. 2.

### Upstream molecular regulation of AURKA

There is overwhelming evidence of overexpression and gene amplification of AURKA in a wide range of cancers. The underlying mechanisms for AURKA upregulation in cancer include gene amplification, gene mutation, microRNA regulation, transcriptional or posttranscriptional modification, and others. Here, we summarize the molecules that positively or negatively regulate AURKA through interactions **(**Table 1**)**.

**Table 1:** Upstream molecules that regulate AURKA

**Positive regulators of AURKA**			
**Names**	**Functions**	**Mechanisms**	**Ref**
FOXM1	Activates AURKA expression at the transcriptional level	FOXM1 binds directly to AURKA promoter to activate AURKA expression.	[1]
ARID3A	Promotes AURKA transcription	Binds to AURKA promoter.	[2]
PUF60	Promotes AURKA transcription	Binds to AURKA promoter.	[3]
E4TF1	Promotes AURKA transcription	Binds to positive regulatory element of AURKA promoter.	[4]
TRAP220/MED1	Promotes AURKA transcription	It binds between the transcription machinery and the GABPα subunit at a region between − 169 and − 98 of AURKA promoter.	[5]
EGFR/ STAT5	Promotes AURKA transcription	EGF induces recruitment of nuclear EGFR and STAT5 to the AURKA promoter.	[6]
β-catenin/ TCF4	Promotes AURKA transcription	Binds to AURKA promoter and enhances AURKA promoter activity.	[7]
HnRNPQ1	Increases the translational efficiency of AURKA mRNA	Enhances the recruitment of ribosomes to those regions of AURKA 5 ′-UTRs.	[8]
NEDD9	Stabilizes AURKA protein expression and increases AURKA activity	Protects AURKA from binding cdh1; Stimulates AURKA autophosphorylation at Thr288.	[9]
TPX2	Ehances AURKA stability and activity	Interaction between AURKA and TPX2 and disassociation from cdh1 is required for protecting AURKA from degradation; Stimulates autophosphorylation and autoactivation of AURKA.	[10, 11] [12]
PUM2	Promotes AURKA stability and activity	Protects AURKA from cdh1-mediated degradation; Increases p-Histone-H3 levels.	[13]
LIMK2	Inhibits AURKA degradation	Association of LIM domains with AURKA is sufficient for AURKA stabilization.	[14]
Twist	Inhibits AURKA degradation	Ubiquitin-proteosomal degradation pathway.	[15]
ALDH1A1	Inhibits AURKA degradation	Ubiquitin-proteosomal degradation pathway.	[16]
YBX1	Inhibits AURKA degradation	Ubiquitin -proteosomal degradation pathway.	[17]
USP2a	Inhibits AURKA degradation	Removes ubiquitin from AURKA.	[18]
PKC	Increases AURKA activity	Phosphorylates AURKA at Thr287, which augments interaction with TPX2.	[19]
PNUTS	Increases AURKA activity	Blocks PP1-dependent dephosphorylation of AURKA.	[20]
BuGZ	Increases AURKA activity	Zinc figers in BuGZ directly bind to the kinase domain of AURKA and stimulates autophosphorylation at Thr288.	[21]
RASSF1A	Increases AURKA activity	Stimulates AURKA autophosphorylation at Thr288.	[22]
IPP2	Increases AURKA activity	Ability to activate MBP is enhanced through inhibition of PP1. No increase in p-Thr288.	[23]
PAK1	Increases AURKA activity	Phosphorylates AURKA at Thr288 and Ser342 sites in the activation loop.	[24]
Ajuba	Increases AURKA activity	Stimulates AURKA autophosphorylation at Thr288 and kinase activity toward histone H3.	[25]
KCTD12	Increases AURKA activity	Stimulates AURKA autophosphorylation at Thr288.	[26]
**Negative regulators of AURKA**			
**Names**	**Functions**	**Mechanisms**	**Ref**
INI1/hSNF5	Represses AURKA transcription	Associates with AURKA promoter.	[27]
ARID1A	Represses AURKA transcription	Associates with AURKA promoter.	[28]
SIX3	Represses AURKA transcription	Associates with AURKA promoter.	[29]
MCPIP1	Inhibits AURKA transcription	Destabilizes AURKA mRNA	[30]
Cdh1	Induces AURKA degradation	Cdh1-APC/C-ubiquitin-proteasome pathway.	[31]
NQO1	Induces AURKA degradation	NQO1 competes with TPX2 for binding to AURKA.	[32]
SMAD4	Induces AURKA degradation	Ubiquitin -proteosomal degradation pathway.	[33]
RPL3	Induces AURKA degradation	Depends on PRL-3-mediated dephosphorylation of FZR1 and assembly of the APC/C^FZR1^ complex.	[34]
IKK2	Induces AURKA degradation	IKK2 phosphorylation of AURKA targets it for β-TRCP-mediated proteasomal degradation.	[35]
AURKAIP1	Induces AURKA degradation	Interaction with AURKA is essential for degradation.	[36] [37]
VHL	Induces AURKA degradation	VHL recognition of AURKA occurs independent of prolyl hydroxylation and results in multi-monoubiquitination.	[38]
PTPRD	Induces AURKA degradation	Dephosphorylates tyrosine residues in AURKA.	[39]
PHLDA1	Induces AURKA degradation	Ubiquitin-proteosomal degradation pathway.	[40]
PTTG1	Inhibits AURKA activity	Attenuates AURKA autophosphorylation at Thr288 and p-Histone-H3 level.	[41]
Gadd45a	Inhibits AURKA activity	Attenuates AURKA ability to phosphorylate MBP.	[42]
PP1	Inhibits AURKA activity	Dephosphorylates AURKA and abolishes kinase activity.	[43]
GSK-3β	Inhibits AURKA activity	Phosphorylates AURKA on S290/291, leading to autophosphorylation of serine 349.	[44]

Multiple myeloma SET domain protein (MMSET); Forkhead box subclass M1 (FOXM1); Human Pumilio homology protein 2 (PUM2); LIM-domain kinase-2 (LIMK2); Aldehyde dehydrogenase 1 (ALDH1A1); Y-box binding protein-1 (YBX1); Protein kinase C (PKC); Phosphatase 1 nuclear targeting subunit (PNUTS); RAS-association domain family 1, isoform A (RASSF1A); Protein phosphatase inhibitor-2(IPP2); P21-activated kinase 1 (PAK1); Potassium channel tetramerization domain containing 12 (KCTD12); Nicotinamide adenine dinucleotide(P) H quinone oxidoreductase 1 (NQO1); Phosphatase of Regenerating Liver-3 (RPL3); IκB kinase 2 (IKK2); Aurora-A Kinase interacting protein (AURKAIP1); Von Hippel-Lindau (VHL); Protein tyrosine phosphatase receptor delta (PTPRD); Pleckstrin homology-like domain family A member 1(PHLDA1); Pituitary tumor transforming gene 1 (PTTG1); Protein Phosphatase 1 (PP1); Glycogen synthase kinase 3 beta (GSK-3β); monocyte chemoattractant protein-induced protein 1 (MCPIP1); poly(U) binding splicing factor 60 (PUF60); SIX homeobox 3 (SIX3); AT-rich interactive domain 1A (ARID1A); AT-rich interaction domain 3A (ARID3A)

#### Positive regulators of AURKA

##### Transcriptional regulation of AURKA

Initially, AURKA function is regulated at the transcriptional level. In breast cancer stem cells, nuclear AURKA is recruited by FOXM1 and binds to the FOXM1 promoter to transactivate its expression, while FOXM1 activates AURKA expression at the transcriptional level in a similar manner [1]. The positive feedback signaling loop between AURKA and FOXM1 is crucial for breast cancer stem cell self-renewal. One study has reported that the transcription of AURKA is positively regulated by E4TF1, which is a ubiquitously expressed ETS family protein [4]. Another study has indicated that EGF-induced AURKA expression depends on the interaction of nuclear EGFR and STAT5 [6]. EGFR associated with STAT5 binds to the AT-rich sequence of AURKA and subsequently increases AURKA transcriptional activity [6]. ARID3A (AT-rich interaction domain 3A) is a transcriptional factor. In colorectal cancer cells, ARID3A can bind with the AURKA promoter region and promote AURKA expression [2]. As a nucleic acid-binding protein, PUF60 contributes to malignant phenotypes of bladder cancer through binding to AURKA promoter and activating AURKA transcription [3]. The TRAP220/MED1 complex [5] and β-catenin/TCF4 complex [7] also directly bind to the AURKA promoter to enhance AURKA transcriptional activity.

##### Translational regulation of AURKA

AURKA is identified as a target protein of HnRNP Q1 by RNA-immunoprecipitation assay following next-generation sequencing. HnRNP Q1 enhances the translational efficiency of AURKA mRNA by interacting with the 5′-UTR of AURKA mRNA through its RNA-binding domains [8]. More importantly, this regulation mechanism is vital for the pro-proliferative properties of HnRNP Q1in colorectal cancer.

##### Regulators promoting AURKA activity

Posttranslational regulation of AURKA is vital for AURKA autophosphorylation and kinase activity. Among the proteins that interact with and activate AURKA, some are well-established activators, such as Ajuba, TPX2, NEDD9 and PUM2. The LIM protein Ajuba efficiently stimulates AURKA autophosphorylation at Thr288 and increases kinase activity toward histone H3 in the late G2 phase [25]. Both the LIM-2 and LIM-3 domains of Ajuba mediate the interaction with the N-terminus of AURKA, and the Ajuba-AURKA complex induces mitotic entry and progression of cell division [25]. Furthermore, activation of AURKA is also stimulated by TPX2. The N-terminal domain of TPX2 binds to AURKA and protects AURKA from dephosphorylation according to experimental and structural analyses [10, 11]. TPX2 primarily exists in an inhibitory complex along with importin α/β at the onset of mitosis, and it is immediately released by Ran-GTP after nuclear envelope breakdown to bind to AURKA and stimulate AURKA autophosphorylation at Thr288. Two other kinases, PAK1 and PKC, directly phosphorylate AURKA and then increase AURKA activity [19, 24]. Other molecules also modulate AURKA activity, such as PNUTS [20], BUGZ [21], RASSF1A [22], IPP2 [23] and KCTD12 [26].

##### Regulators stabilizing AURKA protein expression

Abnormally upregulated AURKA in cancers is always stabilized by other molecules. Protein kinases such as LIMK2 are associated with AURKA through LIM domains, and this interaction is responsible for AURKA stabilization [14]. TPX2 protects AURKA from degradation both in interphase and in mitosis in a cdh1-dependent manner [12]. Likewise, NEDD9 [9] and PUM2 [13] not only stimulate autophosphorylation and autoactivation of AURKA but also stabilize AURKA protein expression through disassociation from cdh. AURKA protein stability is also maintained by Twist [15], ALDH1A1 [16], YBX1 [17] and the deubiquitinase USP2a [18] through ubiquitin-proteosomal degradation pathway.

#### Negative regulators of AURKA

Although many proteins determine the active state of AURKA to a great extent, negative AURKA regulators that tightly control AURKA expression or activity exist. These regulators are usually tumor suppressors, and inhibition of AURKA is one of the mechanisms explaining their tumor-suppressive functions.

##### Transcriptional regulation of AURKA

INI1/hSNF5 is a core component of the mammalian chromatin-remodeling SWI/SNF complex, which regulates the transcription of target genes. In rhabdoid tumor (RT) cells and normal fibroblast cells, INI1/hSNF5 complex associates with the AURKA promoter and represses AURKA transcription. This regulation is dependent on cell type because in non-RT cells such as Jurkats, CEMX-174, HeLa and SF268, downregulation of INI1/hSNF5 had either no effect or a slight decrease in AURKA [27]. ARID1A, a component of the SWI/SNF chromatin remodeling complex, occupies the AURKA gene promoter to negatively regulate its transcription [28]. SIX3, a member of the sine oculis homeobox transcription factor family, suppresses the transcription of both AURKA and AURKB by directly binding with their promoters in astrocytoma [29]. Apart from the regulation of AURKA transcription through interaction with AURKA promoter, it was reported that ribonuclease MCPIP1 destabilized AURKA mRNA [30]. A highly conserved 95-base region in AURKA 3′-UTR was required for MCPIP1-dependent cleavage of the AURKA transcript [30].

##### Regulators reducing AURKA activity

GSK-3β interacts with AURKA and phosphorylates AURKA at Ser290/291 in vitro, after which autophosphorylation occurs at Ser349, which is an AURKA activity-inhibiting phosphorylation site [44]. Gadd45a is a stress gene that is highly induced by a variety of genotoxic agents. Interaction between Gadd45a and AURKA has been shown to strongly inhibit AURKA kinase activity and antagonize AURKA-induced centrosome amplification [42]. PTTG1 is a transforming gene highly expressed in several cancers. One study has indicated that PTTG1 represses AURKA autophosphorylation, inhibits phosphorylation of histone H3 and results in abnormally condensed chromatin [41]. Another study has shown that the phosphatase PP1, but not PP2, dephosphorylates AURKA and abolishes AURKA kinase activity [43].

##### Regulators promoting AURKA protein degradation

Apart from AURKA activity, AURKA protein expression is tightly controlled as well. During the process of mitosis, IKK2 acts as an antagonist of AURKA. Phosphorylation of AURKA by IKK2 targets it for β-TRCP-mediated degradation and serves to maintain appropriate levels of AURKA to assure proper bipolar spindle assembly and mitotic progression [35]. AURKAIP1, an AURKA-interacting protein, is involved in the degradation of AURKA through a proteasome-dependent pathway [36]. A mechanistic study has revealed that AURKAIP1-mediated AURKA degradation is dependent on antizyme1 (Az1). AURKAIP1 enhances the ability of Az1 to bind to AURKA in order to promote proteasomal localization and subsequent degradation [37]. Cdh1 is a WD40 repeat protein serving as an anaphase-promoting complex/cyclosome (APC/C) coactivator. AURKA degradation is dependent on Cdh1 in vivo*,* and AURKA is targeted for proteolysis through distinct structural features of its destruction box, its KEN box motifs and its kinase activity [31]. VHL is an E3 ligase that multi-monoubiquitinates AURKA in quiescent cells and targets it for proteasome-mediated degradation under both normoxic and hypoxic conditions [38]. Phosphatase PRL-3 enhances AURKA ubiquitination and degradation in colorectal cancer [34]. Destabilization of AURKA by PRL-3 requires PRL-3-mediated dephosphorylation of FZR1 and assembly of the APC/CFZR1 complex [34]. PTPRD is a protein tyrosine phosphatase and a tumor suppressor. It destabilizes the AURKA protein by dephosphorylating tyrosine residues in AURKA, leading to downstream destabilization of the MYCN protein [39]. NQO1 [32], SMAD4 [33] and PHLDA1 [40] are also tumor suppressors mediating AURKA protein degradation. NQO1 competes with TPX2 for binding to AURKA and inhibits excessive increases in AURKA protein levels, thereby suppressing the generation of aneuploidy in irradiated cells [32]. The tumor suppressor SMAD4 interacts with AURKA and inhibits the expression of AURKA via proteasomal degradation which is independent of TGFβ signaling [33].

### Downstream targets of AURKA

Based on the high expression and significance of AURKA in multiple types of tumors, it is crucial to discover the mechanism of action for AURKA in cancer. As a serine/threonine protein kinase, AURKA is reported to interact with numerous proteins, including tumor suppressors and oncogenes, to promote carcinogenesis, as shown in Table 2**.**

**Table 2:** AURKA downstream substrates

Substrates	Phosphorylation sites	Experimental methods	Functional significance	Ref
YBX1	T62, S102	Consensus motif, in vitro kinase assay, autoradiography	Increases YBX1 protein stability, regulate EMT, CSC and chemoresistance.	[17]
LDHB	S162	In vitro kinase assay, MS analysis, point mutation, anti-phosphoserine	Promotes glycolysis and biosynthesis; Promotes tumor growth.	[45]
Merlin	S518	In vitro kinase assay, specifc antibody	Weakens merlin interaction with tubulin.	[46]
RPS6KB1	T389	P-RPS6KB1 (T389) specifc antibody	Reduces survival of KRAS-mutant gastrointestinal cancer cells	[47]
CENP-A	S7	In vitro kinase assay, truncation mutants, autoradiography	Regulates mitosis especially kinetochore function.	[48]
LKB1	S299	In vitro kinase assay, MS analysis, point mutation, autoradiography	Impairs LKB1 interaction with and phosphorylation of AMPK.	[49]
KCTD12	S243	Point mutation, IP, anti-phosphoserine	Promotion of cancer cell proliferation and tumorigenesis.	[26]
CHIP	S273	Point mutation, in vitro kinase assay, autoradiography	Promotes AR degradation via the proteasome pathway.	[50]
ALDH1A1	T267, T442, T493	Consensus motif, point mutation, in vitro kinase assay, autoradiography	Regulates protein stability, EMT and CSC phenotypes.	[16]
Twist	S123, T148, S184	Consensus motif, point mutation, in vitro kinase assay, autoradiography	Regulates protein stability, subcellular localization, EMT, the CSC phenotype and drug resistance.	[15]
YAP	S397	In vitro kinase assay, truncation mutants, MS, consensus motif	Enhances transforming ability.	[51]
TACC3	S558	MS, point mutation, in vitro kinase assay, autoradiography	Regulates TACC3 localization to centrosomes and proximal mitotic spindles.	[52]
HDM2	S166	In vitro kinase assay, specifc antibody	NA	[53]
β-catenin	S552, S675	In vitro kinase assay, truncation mutants, autoradiography	Increases its stability and transcriptional activity	[54]
ERα	S167, S305	In vitro kinase assay, truncation mutants, autoradiography	Leading to increase in ERα DNA-binding and transcriptional activity.	[55]
BimEL	S93/94/98	Consensus motif, in vitro kinase assay, specifc antibody	NA	[56]
GSK-3β	S9	In vitro kinase assay, specifc antibody	Leading to accumulation and activation of the β-catenin/TCF complex.	[57]
PLK1	T210	In vitro kinase assay, specifc antibody	Centrosomal organization.	[58]
IκBα	S32, S36	Specifc antibody	NFκB activation.	[59]
VHL	S72	In vitro kinase assay, MS, autoradiography	NA	[60]
PHLDA1	S98	In vitro kinase assay, consensus motif, autoradiography	Negatively regulates PHLDA1 by promoting PHLDA1 degradation.	[40]
Histone H3	S10	In vitro kinase assay, autoradiography	Involved in the initiation of mitosis.	[61]
YY1	S365	In vitro kinase assay, truncation mutants, consensus motif, autoradiography	Abolishes its DNA binding activity and transcriptional activity.	[62]
SDCBP	S131, T200	In vitro kinase assay, consensus motif, autoradiography, anti-phospho-serine/threonine	Stabilizes SDCBP protein and regulates its oncogenic function.	[63]
SOX2	S220, S251	In vitro kinase assay, MS	Maintains the ratio of stem-cell like cells.	[64]
CPAP	S467	In vitro kinase assay, truncation mutants, MS, autoradiography	Is required for the integrity of the spindle pole during mitosis.	[65]
RalA	S194	In vitro kinase assay, consensus motif, autoradiography	Enhances cell migration and anchorage-independent growth.	[66]
CDC25B	S353	MS, in vitro kinase assay, specifc antibody	Control of the onset of mitosis.	[67]
NDEL1	S251	MS, in vitro kinase assay, autoradiography	Centrosomal maturation, separation, and TACC3 recruitment.	[68]
ASAP	S625	MS, in vitro kinase assay, autoradiography	Bipolar spindle assembly.	[69]
MBD3	S24	In vitro kinase assay, autoradiography, consensus motif	NA	[70]
RASSF1A	T202, S203	Consensus motif, in vitro kinase assay, autoradiography	Induces M-phase cell cycle arrest.	[71]
P53	S215, S315 S106	Consensus motif, in vitro kinase assay, autoradiography	S215: Abrogates DNA binding and transactivation activity; S315: Inactivates p53 by enhancing its proteolytic degradation. S106: Inhibit the interaction of p53 with MDM2 and prolong the half-life of p53.	[72] [73] [74]
HURP	S627, S725, S757, S830	In vitro kinase assay,autoradiography, MS	Regulates HURP stability and exhibits serum-independent growth.	[75]
PP1	NA	In vitro kinase assay,autoradiography	Inhibits PP1 activity at mitosis.	[43]
P73	S235	Consensus motif, in vitro kinase assay, autoradiography	Abrogates its transactivation function; Regulates subcellular localization.	[76]
TPX2	S121, S125	Consensus motif, in vitro kinase assay, autoradiography	Maintains metaphase spindle length by regulating the microtubules flux.	[77]
Lats2	S83	In vitro kinase assay, truncation mutants, autoradiography	Regulates centrosomal localization.	[78]
LIMK2	S283, T494, T505	Consensus motif, in vitro kinase assay, autoradiography	Inhibits LIMK2 degradation and increases LIMK2 kinase activity.	[14]

Y-box binding protein-1 (YBX1); Lactate dehydrogenase B (LDHB); Ribosomal protein S6 kinase B1 (RPS6KB1); Centromeric protein A (CENP-A); Liver kinase B1 (LKB1); Potassium channel tetramerization domain containing 12 (KCTD12); C-terminus of HSP70-interacting protein (CHIP); Aldehyde dehydrogenase 1 (ALDH1A1); Yes-associated protein (YAP); Glycogen synthase kinase 3 beta (GSK-3β);Polo-like kinase-1 (PLK1); Pleckstrin homology-like domain family A member 1(PHLDA1); Yin Yang 1 (YY1); Syndecan binding protein (SDCBP); Sex-determining region Y (SRY)-Box 2 (SOX2); Centrosomal P4.1-associated protein (CPAP); Ral small GTPase (RalA); Neurodevelopment protein 1-like 1 (NDEL1); ASter-Associated Protein (ASAP); RAS-association domain family 1, isoform A (RASSF1A); Hepatoma upregulated protein (HURP); Protein Phosphatase 1 (PP1); Large tumor suppressor 2 (Lats2); LIM-domain kinase-2 (LIMK2); Mass spectrometry (MS); NA: not available

#### AURKA substrates regulating mitosis

AURKA is involved in the regulation of spindle-associated events during early mitosis. Many of the substrates regulated by AURKA coordinate with AURKA to control mitotic progression, and aberrant expression of AURKA in a variety of human cancers has been linked with mitotic defects. Phosphorylation of histone H3 is a crucial event for the onset of mitosis. AURKA physically interacts with the histone H3 tail and efficiently phosphorylates Ser10 both in vitro and in vivo [61]. NDEL1 phosphorylation by AURKA at the Ser251 site is essential for centrosomal separation and centrosomal maturation. After phosphorylation, NDEL1 displays high affinity for the mitotic protein TACC3, mediating TACC3 recruitment to the centrosome [68]. TACC3 is another substrate of AURKA that is localized to mitotic spindles and proximal mitotic spindles after phosphorylation at Ser558 [52]. The NDEL1-TACC3 protein complex activated and initiated by AURKA plays a significant role in centrosome maturation and separation during mitosis. Another centrosome-associated protein, CPAP, directly interacts with AURKA and is phosphorylated by AURKA at Ser467 to maintain the integrity of the spindle pole [65]. ASAP is also a spindle-associated protein, deregulation of which induces severe mitotic defects. After phosphorylation at Ser625 by AURKA, ASAP localizes to centrosomes from late G2 to telophase and around the midbody during cytokinesis [69]. The AURKA activator TPX2 is an AURKA substrate with phosphorylation sites at Ser121 and Ser125. Phosphorylation of TPX2 by AURKA is required for establishment of normal spindle length and interaction with cytoplasmic linker-associated protein 1 [77]. PLK1 is an essential mitotic kinase regulating multiple aspects of the cell division process, and activation of PLK1 requires phosphorylation at Thr210 in the T-loop of the PLK1 kinase domain. It has been reported that AURKA is responsible for the Thr210 phosphorylation of PLK1, which is required for checkpoint recovery [58]. Another study has demonstrated that the phosphatase CDC25B is phosphorylated by AURKA at the Ser353 site to contribute to the G2-M transition [67]. CENP-A, a well-conserved variant of histone H3, is phosphorylated by AURKA at Ser7, which is required for the concentration of AURKB at inner centromeres and for kinetochore function [48].

#### AURKA substrates acting as functional oncogenes

Some AURKA substrates, such as GSK-3β, β-catenin, Twist, ERα, IκBα, and YAP, participate in crucial oncogenic signaling. AURKA and GSK-3β exist in a complex, and a significant increase in the phosphorylation of GSK-3β at Ser9 has been observed following overexpression of AURKA [57]. Furthermore, AURKA inhibits the degradation of β-catenin, a known substrate of GSK-3β, by phosphorylating β-catenin at the Ser552 and Ser675 sites [54]. This phosphorylation also regulates β-catenin nuclear localization and transcriptional activity toward its target genes [54]. Research has shown that AURKA phosphorylation of Twist at Ser123, Thr148 and Ser184 facilitates Twist-mediated promotion of EMT and chemoresistance in pancreatic cancer cells [15]. In addition, AURKA interacts with ERα and phosphorylates it at Ser167 and Ser305, leading to an increase in the DNA-binding ability of ERα and the transcriptional activity of ERα toward its target cyclin D1 [55]. More interestingly, elevated expression of AURKA predicts poor survival in ERα-positive but not in ERα-negative breast cancers [55]. Regarding the pathway by which AURKA regulates NF-κB signaling, a mechanistic study has revealed that IκBα phosphorylation by AURKA promotes its degradation, thus activating the NF-κB pathway [59]. YAP is the main downstream effector of the Hippo pathway. AURKA-mediated phosphorylation of YAP at Ser397 is crucial for YAP-mediated transcriptional activity and transformation in triple-negative breast cancer cells [51].

SOX2 and YBX1 are oncogenic transcription factors phosphorylated by AURKA. After phosphorylation, SOX2 is able to maintain the ratio of stem cell-like cells, while YBX1 is stabilized and enhances EMT, stem cell formation and chemoresistance [17, 64]. LDHB is a subunit of the tetrameric enzyme LDH that catalyzes the interconversion between pyruvate and lactate. Phosphorylation of LDHB by AURKA at Ser162 amplifies its activity in reducing pyruvate to lactate, thus promoting glycolysis and biosynthesis and promoting tumor growth [45]. Recently, our research has indicated that phosphorylation of the scaffold and oncogenic protein SDCBP by AURKA maintains its protein stability and pro-proliferative functions [63]. Furthermore, the ability of SDCBP to bind to its partners, including EGFR, SRC and FAK, is attenuated when the phosphorylation sites are inactivated [63]. Another unique substrate of AURKA is HURP, which is phosphorylated at four serine positions [75]. HURP protein stability and serum-independent growth are enhanced after phosphorylation [75].

RPS6KB1, a mitogen-activated serine/threonine protein kinase, is activated in human malignancies. Activation of RPS6KB1 occurs through phosphorylation by AURKA at the Thr389 position, which is important for promoting cell proliferation and survival [47]. LIMK2 is a crucial oncogenic regulator with serine/threonine protein kinase activity. AURKA regulates LIMK2 kinase activity, subcellular localization and protein levels by directly phosphorylating LIMK2 at Ser283, Thr494 and Thr505 [14]. The small GTPase RalA is also a target of AURKA; phosphorylation of RalA at Ser194 enhances cell migration and anchorage-independent growth [66]. ALDH1A1 is an AURKA substrate enzyme whose phosphorylation by AURKA at Thr267, Thr442 and Thr493 regulates ALDH1A1 protein stability, enhancing the role of this protein in the process of EMT [16].

#### AURKA substrates acting as tumor suppressors

P53 is one of the most important substrates of AURKA. It has been reported that AURKA phosphorylates p53 at Ser315, after which p53 is destabilized and the G2-M transition is enhanced [72]. However, Ser106 residue phosphorylation by AURKA has the opposite effect. After phosphorylation, the interaction of p53 with MDM2 is inhibited and the p53 protein expression is stabilized [73]. Another study has revealed that the p53 Ser215 site is phosphorylated by AURKA. P53 DNA binding ability and transactivation activity are inhibited after phosphorylation and p53 tumor suppressor activity is inhibited by AURKA [74]. RASSF1A, initially identified as a microtubule- and centrosome-associated protein, is a scaffold protein with tumor-suppressive function. Phosphorylation of RASSF1A by AURKA at Ser203 and Thr202 removes the ability of RASSF1A to interact with microtubules and induce M-phase cell cycle arrest [71]. PHLDA1, a novel p53 target, can repress the Akt signaling pathway. AURKA directly phosphorylates PHLDA1 at Ser89, which results in degradation of PHLDA1 [40]. Another novel substrate of AURKA with tumor-suppressive function is LKB1. Phosphorylation of LKB1 at Ser299 causes LKB1 to dissociate from AMPK, resulting in impairment of the AMPK signaling pathway and facilitating non-small-cell lung cancer (NSCLC) growth and migration [49]. Merlin suppresses tumor development through distinct mechanisms and is a substrate of AURKA that is phosphorylated at its main regulatory site, Ser518, during mitosis [46]. Another AURKA substrate acting as a tumor suppressor is Lats2. Phosphorylation of Lats2 by AURKA at the Ser83 site regulates its centrosomal localization [78]. This process may be important for Lats2 to suppress tumorigenicity and to inhibit cell proliferation via centrosomal regulation.

#### Other AURKA substrates

Several AURKA substrates exhibit multiple and counteracting functions in cancer development. YY1 and P73, as transcription factors, have been shown to bind hundreds of DNA sites and to regulate a very large number of target genes with a wide range of functionalities. Once YY1 is phosphorylated by AURKA at Ser365, its DNA-binding activity and transcriptional activity are abolished [62]. Furthermore, AURKA phosphorylation of p73 at Ser235 eliminates the p73 transactivation function in both DNA damage-induced cell death and mitotic spindle assembly checkpoint pathways [76]. Another multifunctional protein is the ubiquitin ligase CHIP, which has been shown to be a regulator of oncogenic pathways or tumor-suppressive pathways. AURKA-mediated phosphorylation of CHIP at Ser273 promotes androgen degradation in castration-resistant prostate cancer [50]. KCTD12 also exhibits dual and opposite functions in cancer. Phosphorylation by AURKA at Ser243 may account for the cancer-promoting effects of KCTD12 [26].

MBD3 [70], HDM2 [53], PP1 [43], VHL [60] and BimEL [56] are also phosphorylated by AURKA, but their subsequent specific functions remain to be revealed. Notably, some proteins, including ALDH1A1, Twist, YBX1, KCTD12, RASS1A, PHLDA1, PP1, TPX2, LIMK2 and VHL, usually form negative or positive feedback loops with AURKA.

### Signaling pathways involving AURKA-interacting proteins

**Fig. 3:**
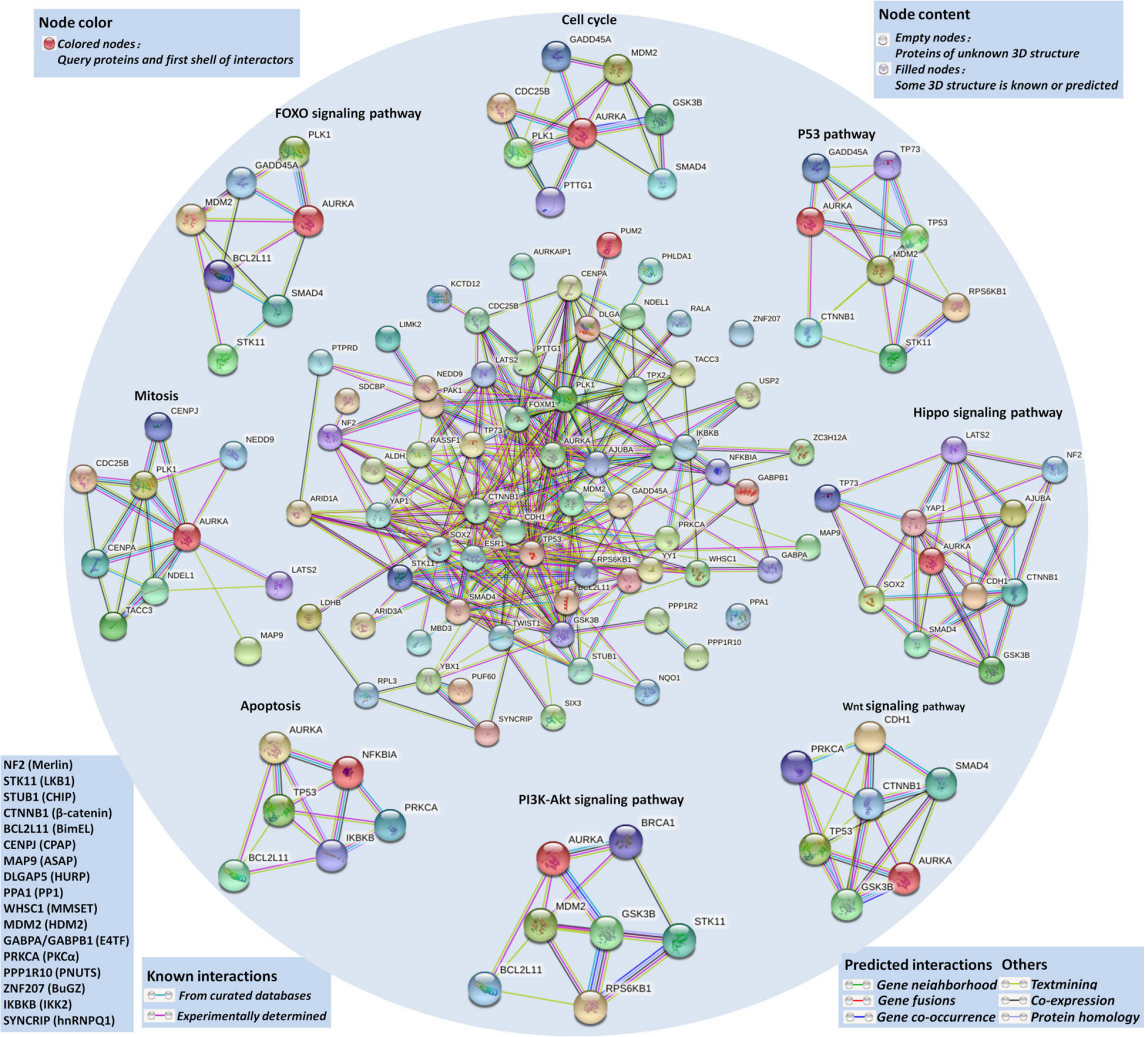
AURKA interactome and related signaling pathways. The interactome in the center is obtained through STRING database based on AURKA-interacted proteins mentioned in Table 1 and Table 2. The interactome around are enriched pathway proteins. The left bottom literal statements are the alternative names of the molecular

AURKA has been identified to regulate many signaling pathways, such as the PI3K/Akt, mTOR, β-catenin/Wnt and NF-κB pathways, and tumorigenesis requires interactions among multiple signaling pathways. We obtained an interactome network using the STRING database based on the AURKA-interacting proteins mentioned in the previous section **(**Fig. 3**)**. Then, we performed Kyoto Encyclopedia of Genes and Genomes (KEGG) pathway and Gene Ontology (GO) enrichment analyses of the signaling pathways. The results indicated that AURKA-related proteins are involved in the processes of mitosis, cell cycle progression and apoptosis. Furthermore, these proteins are directly or indirectly associated with key molecules in crucial signaling pathways such as the Hippo pathway, the p53 pathway, the PI3K-Akt pathway, the FOXO pathway and the Wnt pathway. Most importantly, AURKA is involved in all of these cancer-related pathways, suggesting the significance of AURKA in these processes and pathways.

### Pharmacologic targeting of AURKA in cancer therapy

A series of molecules have been demonstrated to be able to inhibit AURKA activity. Although the majority also exerts effects on other members of the Aurora kinase family or even on other kinases, there is enough evidence to make some of them potent targets for cancer therapy both in vitro and in vivo in preclinical or clinical evaluations **(**Table 3**and** Table 4**).**

**Table 3:** AKIs in preclinical studies

Compound names	Structures	Targets (IC50)	Cell-based potency (IC50/EC50/GI50)	Animal models (type; concentration; efficiency)	Ref
CYC3		AURKA (0.033 μM))	IC50: MIA PaCa-2 (1.1 μM) PANC-1 (2 μM)	NA	[79]
AKI603		AURKA (12.3 nM)	NA	Epirubicin resistant MCF-7 cell xenograft; mice were treated intra-gastrically every day with 50 mg/kg AKI603; tumor volume and tumor weight were significantly reduced.	[80]
BPR1K0609S1(BP)		AURKA (43 nM)	HCT116 (400 nM)	Parental and BP-resistant HCT116 Puma(−), Bax(−), Chk2(−) and p53(−) cells were transplanted into nude mice; these BP-resistant cells did not show faster tumor development compared to their parental cells, respectively.	[81, 82]
LDD970		AURKA (0.37 μM)	IC50: HT29 (4.22 μM)	NA	[83]
MK-8745		AURKA (0.6 nM)	NA	HCT116, HCT116 p53(−), HCT116 Puma(−), HCT116 p21(−) and HCT116 Chk2(−) xenografts; MK-8745 (800 nM) was directly s.c. injected daily; HCT116 p53(−) tumorigenesis was weakly inhibited, HCT116 Puma(−), HCT116 p21(−), HCT116 Bax(−) and HCT116 Chk2(−) cells was inhibited with MK-8745.	[84, 85]
LY3295668		AURKA (< 1 nM)	55 out of 80 cell lines displayed sensitivity (IC50 < 1 μM) to LY3295668 with an average IC50 of 0.048 μM	1. NCI-H446 xenograft model; 50 mg/kg (s.c), (BIDX7, rest 14) X 2, (BIDX14, rest 7) X 2, or (BIDx21) X 2 schedule; produced significant tumor growth inhibition. 2. PDX; 50 mg/kg BIDX28 showed 97.2% of tumor growth inhibition.	[86]
BPR1K653		AURKA (124 nM) AURKB (45 nM)	IC50: A549 (9 nM) HT29 (12 nM) OECM-1 (135 nM) HONE-1 (11 nM) KB (12 nM) NTUB1 (8 nM) MV4–11(5 nM) IM9 (4 nM)	Cervical cancer KB xenograft; 15 mg/kg through intravenous injection for two weeks; 73% decrease in tumor volume.	[87]
TY-011		AURKA (102.1 nM) AURKB (93.9 nM)	IC50: SNU-16 (0.09 μM) MKN-45 (0.13 μM) MGC-803 (0.19 μM) SGC-7901(0.57 μM) AGS (0.96 μM)	Gastric cancer cell MGC-803 xenograft; TY-011 was orally administered at 3, 6 and 9 mg/kg once a day for 13 days; 64.9, 87.7, 89% inhibition rate for 3, 6 and 9 mg/kg respectively.	[88]
BPR1K871		AURKA (22 nM) AURKB (13 nM) FLT3 (19 nM)	EC50 values ranged from 34 nM to 7 μM in various cancer cells. COLO205 (34 nM) Mia-PaCa2 (94 nM)	Colorectal COLO205 or pancreatic Mia-PaCa2 xenograft; 15 mg/kg intravenous administration of BPR1K871 once a day for two continuous weeks (on days 1–5 and 8–12); TGI was about 90%.	[89]
SCH-1473759		AURKA (≤4 nM) AURKB (≤ 13 nM)	In 51/53 tumor cells IC50 < 100 nM. The mean IC50 was 21 nM. A2780, LNCap, N87, Molt4, K562, and CCRF-CEM with IC50 < 5 nM.	A2780 human tumor xenograft; 5 mg/kg (i.p) bid dosed daily on days 0–16 (TGI = 50%) and 10 mg/kg (i.p) bid dosed intermittently on days 0–4 and 10–14 (TGI = 69%).	[90]
Derrone		AURKA (22.3 μM) AURKB (6 μM)	H1299 (23.8 μM) MCF7 (24.4 μM) Hela (31.2 μM) KPL4 (45.8 μM)	NA	[91]
JNJ-7706621		AURKA (11 nM) AURKB (15 nM) CDK1 (9 nM) CDK2 (4 nM)	IC50 values ranged from 112 to 514 nM in various cancer cell lines while IC50 values ranged from 3.67 to 5.42 μM in normal cells.	A375 xenograft model; JNJ-7706621 was treated i.p using 125 mg/kg 7 on/7 off schedule and the 100 mg/kg QD schedule; TGI values were 93% for both two schedules.	[92]
SAR156497		AURKA (0.6 nM) AURKB (1 nM) AURKC (3 nM)	SAR156497 was active on various tumor cell lines (IC50: 5–500 nM).	HCT116 xenografts; compound was s.c injected in continuous infusion using ALZET micropump at an 8 μL/h flow rate for 48 h and at doses of 25 mg/kg; TGI% = 81%. Note: therapeutic window was narrow.	[93]
R1498		VEGFR2 (25 nM) AURKA (67 nM) AURKB (167 nM)	Mean IC50s were 7.81, 7.55 and 30.07 μM in epatocellular carcinoma, gastric cancer, and nasopharyngeal carcinoma cell lines, respectively.	1. BEL-7402, MGC-803 and SGC-7901 xenografts; 25 mg/kg, twice daily, orally; R1498 showed better TGI% over sorafenib. 2. CNE-2 xenograft; 25 mg/kg, twice-daily, oral gavage; TGI% was 90%. 3. Three PDX model; TGI% reached 73.6–91.6% (25 mg/kg, twice-daily, oral gavage).	[94]
VE-465		AURKA (1 nM) AURKB (26 nM) AURKC (8.7 nM)	Huh-7 (2.01 μM) HepG2 (4.15 μM)	HCC human Huh-7 xenograft; twice a day i.p with VE-465 at 15, 25, and 35 mg/kg for 14 days; the mean tumor volumes were reduced by 59, 59, and 77%, respectively.	[95]
CCT129202		AURKA (0.042 μM) AURKB (0.198 μM) AURKC (0.227 μM)	GI50: Colo205 (0.46 μM) SW620 (0.7 μM) HCT116 (0.35 μM) HT29 (0.5 μM) KW12 (1.2 μM) Hela (0.2 μM) A2780 (0.3 μM) OVCAR8 (1 μM) MV4–11 (0.08 μM)	HCT116 colon carcinoma xenografts; mice were treated i.p. with a single dose of 100 mg/kg /day for 9 days; Significant tumor growth inhibition was observed compared with the vehicle-treated mice (% treated versus control, 57.7; *P* = 0.0355)	[96]
CCT137690		AURKA (0.015 μM) AURKB (0.025 μM) AURKC (0.019 μM) FLT3 (0.0025 μM)	CCT137690 effectively inhibited the growth of human tumor cell lines of different origins with GI50 values ranging from 0.005 to 0.47 Μm.	1. SW620 xenografts; orally at a dose of 75 mg/kg twice a day for 21 days; The treated/control (T/C) ratio was calculated as 42.4% based on final tumor weight. 2. MYCN-driven transgenic mouse model; 100 mg/kg twice daily for 10 days; TGI was observed as early as day 3 and continuous treatment showed significant tumor growth inhibition at day 7 and day 10.	[97, 98]
PHA-680632		AURKA (27 nM) AURKB (135 nM) AURKC (120 nM)	PHA-680632 has potent antiproliferative activity in a wide range of cell types with an IC50 in the range of 0.06 to 7.15 μM.	1. HL60 xenograft; mice were injected i.v. at three dose levels (15, 30, and 45 mg/kg for 5 days); the 45 mg/kg dose resulted in 85% of TGI without signs of toxicity. 2. A2780 xenograft; 60 mg/kg i.v. for 5 days; TGI% = 78%. 3. HCT116 colon carcinoma xenograft; 15 and 30 mg/kg i.p for 12 days; TGI was 75%.	[99]
AKI-001		AURKA (0.004 μM) AURKB (0.005 μM)	HCT116 (0.07 μM) HT29 (0.07 μM) MCF7 (0.1 μM)	HCT-116 xenograft model; Mice were dosed orally QD for 21 days (0, 1, 2.5, 5, or 10 mg/kg); 2.5 (82% TGI) and 5 mg/kg (92% TGI). Note: dosing at 10 mg/kg QD led to unacceptable weight loss, marked bone marrow depletion, and gastrointestinal hypocellularity.	[100]
Reversine		AURKA (400 nM) AURKB (500 nM) AURKC (400 nM)	IC50 values ranged from 100 to 1000 nM of a wide variety of tumor cell lines.	U14 cell xenograft; mice were treated with reversine (10 mg/kg) alone or with aspirin (1 μg/kg), i.p per 3 days; tumor growth was reduced and the mice survived longer in the combination group.	[101]

Tumor growth inhibition (TGI); Intraperitoneal injection (i.p); Subcutaneous (s.c); Intravenous (i.v); Growth inhibition by 50% (GI50); Twice a day (BID); Once a day (QD); NA: not available

**Table 4:** AKIs in clinical development

Drug name	Targets (IC50/Ki value)	Phase of trial	Clinical Trial ID	Patients	Administration	Efficiency	Ref
AMG900	IC50 value: AURKA (5 nM) AURKB (4 nM) AURKC (1 nM)	I	NCT01380756	Acute myeloid leukemia (*n* = 35)	Doses from 15 to 100 mg or doses from 30 to 50 mg, orally, once daily.	CRi = 9% (80% confidence interval: 3, 18%).	[102]
		I	NCT00858377	TPROC (*n* = 29) TNBC (*n* = 14) CRPC (*n* = 12)	40 mg, orally, once daily.	PR = 10.3%for TPROC patients; No responses for TNBC and CRPC.	[103]
AS703569 (R763) (MSC1992371A)	IC50 value: AURKA (4.0 nM) AURKB (4.8 nM) AURKC (6.8 nM)	I	NCT00391521	Solid tumors (*n* = 92)	60–74 mg/m^2^/21-day cycle, orally.	No patients had PR or PR.	[104]
		I	NCT01080664	Hematologic malignancies (*n* = 75)	37 or 28 mg/m^2^/day, orally.	3 patients obtained CR.	[105]
		I	NCT01097512	Solid tumors (*n* = 66)	37 mg/m^2^/day, orally. Combined with standard 1000 mg/m^2^ dose of gemcitabine.	2 patients obtained PR. 5 patients had SD.	[106]
AT9283	IC50 value: AURKA (3 nM) AURKB (3 nM) JAK3 (1.1 nM) JAK2 (1.2 nM) Abl (T315I) (4 nM)	I	NCT00443976	Advanced malignancies (*n* = 35)	40 mg/m^2^/day on days 1, 8 of a 21-day cycle, i.v.	1 patient had PR. 4 patients had SD.	[107]
		I	NCT00522990	R/R leukemia or myelofibrosis (*n* = 48)	108 mg/m^2^/d for 72-h infusion and 40 mg/m^2^/d for 96-h infusion.	2 patients showed benefit.	[108]
		I	CR0708–11	Solid tumors (*n* = 33)	18.5 mg/m^2^/d, i.v.	1 patient had PR.	[109]
		I /II	NCT01431664	Leukemia (n = 7)	9,14.5 or 23 mg/m^2^/day, i.v.	No patients showed responses.	[110]
		II	NCT01145989	R/R multiple myeloma (*n* = 8)	40 mg/m^2^/day or 30 mg/m^2^/day, i.v.	No objective responses.	[111]
BI-847325	IC50 value: AURKA (25 nM) AURKC (15 nM) MEK1 (25 nM) MEK2 (4 nM)	I	NCT01324830	Advanced solid tumors (*n* = 69)	Cumulative dose was 1680 or 2250 mg per 3-week cycle. Orally, once daily.	1 patient had PR. 21 patients had SD.	[112]
CYC116	Ki value: AURKA (8 nM) AURKB (9.2 nM)	I	NCT00560716 (Terminated)	Advanced solid tumors (*n* = 40)	NA	NA	NA
ENMD-2076	IC50 value: AURKA(14 nM) FLT3 (3 nM)	I	NCT00658671	Solid tumors (*n* = 67)	60 to 200 mg/m^2^, Orally, once daily.	2 patients had PR.	[113]
		I	NCT00904787	R/R AML/CML (*n* = 27)	225 mg, 375 mg, 325 mg or 275 mg. Orally, once daily.	1 patient had CRi. 3 patients had MLFS.	[114]
		II	NCT01104675	Ovarian cancer (*n* = 64)	250 mg or 275 mg/d. Orally, once daily.	PFS rate at 6 months was 22%.	[115]
		II	NCT01639248	TNBC (*n* = 41)	250 mg. Orally, once daily.	6-month CBR was 16.7%, 2 PR. 4-month CBR was 27.8%.	[116]
		II	NCT01914510	Ovarian clear cell carcinoma (n = 40)	275 mg (250 mg for patients ≤1.65 m^2^).	3 patients had PR, 26 patients had SD.	[117]
		II	NCT01719744	Advanced soft-tissue sarcomas (*n* = 25)	275 mg. Orally, once daily.	2 patients had PR, 2 patients had SD. CBR was 17% and ORR was 9%.	[118]
		II	NCT02234986	Advanced fibrolamellar carcinoma (n = 35)	150–250 mg. Orally, once daily	1 patient had PR, 20 patients had SD.	[119]
MK-0457 (VX-680, Tozasertib)	Ki value: AURKA (0.6 nM) AURKB (18 nM) AURKC (4.6 nM)	I	NCT02532868	Advanced solid tumors (n = 27)	64 mg/m^2^/h 24-h infusion every 21 days.	12 patients had SD.	[120]
		I/II	NCT00111683	BCR-ABL T315I leukemia (*n* = 77)	40 mg/m^2^/h 5-day infusion or 144 mg/m^2^/h 24-h infusion.	1 patient had CR. 8 patients had hematologic responses.	[121]
		II	NCT00405054	T315I mutant CML and Ph^+^ ALL (*n* = 52)	40 mg/ m^2^/h, 32 mg/ m^2^/h or 24 mg/ m^2^/h 5-day infusion.	8% of patients had major cytogenetic response. 6% achieved unconfirmed complete or partial response.	[122]
MK-5108 (VX-689)	IC50 value: AURKA (0.064 nM)	I	NCT00543387	Advanced or refractory solid tumors (n = 35)	200 or 450 mg/day. Orally, twice daily.	1 patient had PR. 16 patients had SD.	[123]
MLN8054	IC50 value: AURKA (4 nM)	I	NCT00249301	Advanced solid tumors (*n* = 61)	60 mg/day plus methylphenidate or modafinil, four times daily, orally.	3 patients had SD.	[124]
		I	NCT00652158	Advanced solid tumors (*n* = 43)	10-80 mg/day, four times daily, orally.	3 patients had SD.	[125]
PF-03814735	IC50 value: AURKA (5 nM) AURKB (0.8 nM)	I	NCT00424632	Advanced solid tumors (*n* = 57)	Days 1–5 (5-100 mg); or days 1–10 (40-60 mg). Orally, once daily.	19 patients had SD.	[126]
PHA-739358 (Danusertib)	IC50 value: AURKA (13 nM) AURKB (79 nM) AURKC (61 nM)	I	NA	Advanced or metastatic solid tumors (*n* = 50)	45 mg/m^2^ 6-h infusion, 250 mg/m^2^ 3-h infusion. MTD: 330 mg/m^2^, 6-h infusion.	23.7% patients had SD.	[127]
		I	NA	Advanced solid tumors (*n* = 56)	Without G-CSF: 500 mg/m^2^; with G-CSF: 750 mg/m^2^, 24-h infusion.	1 patient had an objective response. 1 patient had 27% tumor regression and 30% CA125 decline.	[128]
		I	2007–004070-18	Accelerated or blastic phase CML, Ph^+^ ALL (*n* = 37)	180 mg/m^2^ 3-h infusion for 7 days in a 14-day cycle.	Responses were observed in four (20%) of the 20 evaluable patients.	[129]
		II	NCT00766324	Prostate cancer (*n* = 88)	330 mg/m^2^ 6-h infusion or 500 mg/m^2^ 24-h infusion.	11 out of 81 (13.6%) patients had SD.	[130]
		II	NA	Solid tumors (*n* = 223)	500 mg/m^2^ 24-h infusion every 14 days.	2 patients had PR.	[131]
SNS-314	IC50 value: AURKA (9 nM) AURKB (31 nM) AURKC (3 nM)		NCT00519662	Advanced solid tumors (*n* = 32)	NA	NA	NA

Complete response (CR); Partial response (PR); Stable disease (SD); Complete response with incomplete count recovery (CRi); Morphologic leukemia-free state (MLFS); Progression free survival (PFS); Granulocyte colony-stimulating factor (G-CSF); Taxane- and platinum-resistant ovarian cancer (TPROC); Triple-negative breast cancer (TNBC); Castration-resistant and taxane- or cisplatin/etoposide–resistant prostate cancer (CRPC); Acute myelogenous leukaemia (AML); Chronic myelogenous leukaemia (CML); Relapsed or Refractory (R/R); Philadelphia Chromosome Positive (Ph^+^); Clinical benefit rate (CBR); Objective response rate (ORR); Not available (NA)

#### Specific AKIs

##### AKIs in preclinical studies

In recent years, several small molecules that selectively target AURKA have been identified with anticancer activity in preclinical studies including LY3295668 [86], BPR1K0609S1 [81, 82], LDD970 [83], MK-8745 [84, 85], AKI603 [80] and CYC3 [79]. The detailed information is shown in Table 3**.**

##### AKIs in clinical studies

Several inhibitors with high specificity for AURKA have been developed, and some of them have shown clinical efficacy in clinical trials. The common dose-limiting toxicity of specific AKIs, including MLN8237 and ENMD-2076, are neutropenia, somnolence and mucisitis.

**MLN8237** is a highly selective small molecule inhibitor of AURKA with an IC50 of 1 nM. MLN8237 was developed as an enhancement of its predecessor, MLN8054, development of which was terminated after phase I studies due to central nervous system side effects, including dose-limiting somnolence [124, 125]. MLN8237 has been shown to inhibit cell proliferation by impairing mitosis, inducing cell cycle arrest and autophagy, and accelerating cancer cell apoptosis and senescence in multiple cancer types [132, 133]. The EMT process is also impeded by MLN8237 treatment in human epithelial ovarian and pancreatic cancer cells [134]. Importantly, MLN8237 significantly increases the sensitivity of tumor cells to chemotherapy drugs or radiation [135, 136]. Mechanistic studies have revealed that MLN8237 induces proteasomal degradation of N-myc in childhood neuroblastoma [137]. In THCA cells, MLN8237 disrupts c-Myc/AURKA complex formation, and c-Myc is a major determinant of MLN8237 responsiveness both in vitro and in vivo [138]. MLN8237 has demonstrated efficacy in cell-derived and patient-derived xenograft (PDX) models of numerous tumor types, including glioblastoma [139], bladder cancer [140], esophageal adenocarcinoma [136], multiple myeloma [132], neuroblastoma [141] and colon cancer [142].

Due to its potent efficiency in preclinical studies, MLN8237 has been tested in clinical trials for multiple cancers and is the only AURKA inhibitor that has proceeded to phase III evaluation. Many phase I and II studies have described the pharmacokinetic and pharmacodynamic properties of MLN8237 in patients with advanced tumors and hematologic malignancies [143–146]. Based on the results of these studies, the recommended phase II dose of MLN8237 is 50 mg twice daily orally for 7 days in 21-day cycles. However, because children experience greater frequencies of myelosuppression and hand-foot-skin syndrome on this schedule, the recommended pediatric phase II dose is 80 mg once daily for 7 days [147]. One phase II trial of MLN8237 in patients with ovarian cancer, fallopian tube cancer, peritoneal carcinoma, acute myelogenous leukemia and high-grade myelodysplastic syndrome showed that MLN8237 has modest single-agent antitumor activity [148]. In a multicenter phase II study, MLN8237 treatment obtained an objective response in 18% of 49 women with breast cancer and 21% of 48 participants with small-cell lung cancer [149]. In another phase II study of MLN8237 in advanced/metastatic sarcoma, occasional responses and prolonged stable disease were observed, and progression-free survival (PFS) was promising [150]. In castration-resistant neuroendocrine prostate cancer patients, those with AURKA and N-myc activation achieve significant clinical benefit from single-agent MLN8237 treatment [151]. Another phase II study has shown that in relapsed or refractory peripheral T-cell NHL (PTCL) patients, MLN8237 has antitumor activity with an overall response rate of 30% [152]. In a recently reported phase III study conducted in patients with PTCL, although MLN8237 did not demonstrate superior efficacy over comparators, it did show antitumor activity and acceptable tolerability and safety [153]. All these encouraging outcomes make MLN8237 a promising agent for cancer treatment.

**ENMD-2076**, a novel, orally bioavailable multitarget inhibitor whose main targets are FLT3 (IC50 = 3 nM) and AURKA (IC50 = 14 nM), exhibits much greater potency against AURKA than against AURKB (IC50 = 350 nM) [154]. Because of its multitarget properties, ENMD-2076 inhibits the growth of a wide range of human solid tumor and hematopoietic cancer cell lines, with IC50 values ranging from 0.025 to 0.7 μM [155]. ENMD-2076 shows antitumor activity in colorectal cancer [154], multiple myeloma [156] and triple-negative breast cancer [157, 158] both in vitro and in vivo. Due to the potent inhibitory effects of ENMD-2076 on cancer cells and xenografts, several phase I/II clinical trials on this compound have been conducted in solid tumors and hematologic malignancies [113–119] **(**Table 4**)**.

**MK-5108** is a novel small molecule that shows robust selectivity for AURKA over AURKB (220-fold greater selectivity) and AURKC (190-fold greater selectivity) [159]. It inhibits the growth of 14 tumor cell lines with IC50 values between 0.16 and 6.4 μM and shows antitumor effects alone or in combination with docetaxel in xenografts [159]. In EOC stem cells, MK-5108 induces cell cycle arrest by affecting the NF-ĸB pathway [160]. MK-5108 also decreases the rate of proliferation and increases intratumoral apoptosis in uterine leiomyosarcoma xenografts [161]. MK-5108 effect has been evaluated in a phase I clinical study as shown in Table 4 [123].

#### Pan Aurora kinase inhibitors

Clinically significant side effects of pan Aurora kinase inhibitors include neutropenia, fatigue, diarrhea and hypertension. Even though the selective AURKA inhibitors might be less toxic than pan-inhibitors, it may also lead to drug resistance more easily, so it is necessary to develop broad Aurora kinase inhibitors to obtain drugs with greater potency for cancer treatment.

##### Inhibitors in preclinical studies

Recently, more than 10 pan-Aurora kinase inhibitors have been designed in preclinical studies. For example, AKI-001 [100], BPR1K871 [89], CCT137690 [97, 98], JNJ-7706621 [92], SAR156497 [93], SCH-1473759 [90] and VE-465 [95] have potent inhibitory effects on Aurora kinase activity with IC50 values< 50 nM. Other Aurora kinase inhibitors with IC50 values> 50 nM against kinase activity, such as BPR1K653 [87], CCT129202 [96], derrone [91], PHA-680632 [99], R1498 [94], reversine [101] and TY-011 [88], have also been tested in preclinical studies, and the preliminary data are shown in Table 3.

##### Inhibitors in clinical studies

**AT9283** exhibits strong activity against several kinases [162]. The ability of AT9283 to inhibit the growth and survival of tumor cells as well as xenografts has been demonstrated in imatinib-resistant BCR-ABL-positive leukemic cells [163], aggressive B-cell lymphoma [164] and medulloblastoma [165]. Several clinical studies have been completed on AT9283 as shown in Table 4 [107–111]. However, there have been no clinical or objective responses in patients in these trials because of the small numbers of patients.

**MK-0457,** a pyrazoloquinazoline compound, inhibits all three Aurora kinases [166] and inhibits FLT-3 and Abl kinases [167]. This compound increases the Bax/Bcl-2 ratio and induces apoptosis in AML cases with high AURKA expression [168]. MK-0457 has been confirmed to show efficiency in anaplastic THCA cells [169], chemoresistant ovarian cancer models [170], myeloma cell lines and primary myeloma cell samples [171], and imatinib-resistant chronic myelogenous leukemia [172]. MK-0457 induces accumulation of cells with ≥4 N DNA content, inhibits cell cycle progression and induces apoptosis of anaplastic THCA cells [169]. In a phase I study conducted in patients with advanced solid tumors, almost half of them attained stable disease, including one patient with advanced ovarian cancer who attained prolonged stable disease for 11 months after receiving 15 cycles of MK-0457 [120]. The activity of MK-0457 was also assessed in two other phase I/II studies, both of which showed promising outcomes in patients with BCR-ABL T315I leukemia [121, 122].

**PHA-739358** exerts strong activity against all three Aurora kinases and cross-reactivity with tyrosine kinases, including FGFR1 and Abl [173]. PHA-739358 exhibits strong antiproliferative activity in BCR-ABL-positive leukemia cells, including those harboring the T315I mutation [174]. The crystal structure of the Abl-T315I-PHA-739358 complex provides a possible structural explanation for the activity of PHA-739358 on the T315I mutation [175]. PHA-739358 also induces cell cycle arrest, apoptosis and autophagy and suppresses the EMT process [176, 177]. More importantly, PHA-739358 shows antimetastasis properties. In one study, PHA-739358 inhibited liver metastases from gastroenteropancreatic neuroendocrine tumors in an orthotopic xenograft model [178]. In another study, PHA-739358 inhibited early-stage bone metastases based on an ex vivo model named the ‘bone-in-culture array’ [179]. Several phase I/II clinical evaluations have been performed on PHA-739358 due to its encouraging antitumor effects [127–131].

Other drugs including AMG900 [102, 103], AS703569 [104–106], BI-847325 [112], CYC116, PF-03814735 [126], and SNS-314 are also in phase I clinical trials.

#### Combination therapy

##### Synergy between AKIs and chemotherapy or radiotherapy

AURKA inhibitors have been shown to have great potential for enhancing the efficacy of multiple established therapeutic agents in both preclinical and clinical studies. AURKA inhibitors combined with docetaxel can produce better therapeutic outcomes than docetaxel alone in mantle cell lymphoma and upper gastrointestinal adenocarcinomas [180–182]. This combination procedure was applied in a phase I clinical trial; in this trial, 20 mg of alisertib twice daily for days 1 to 7 with intravenous docetaxel at 75 mg/m^2^ on day 1 in a 21-day cycle was well tolerated, and the combination regimen demonstrated antitumor activity in various cancer types [183]. Combined treatment with alisertib and paclitaxel has been found to result in more potent inhibition of tumor growth and dissemination than single-agent treatment in an orthotopic xenograft model of EOC [184]. Moreover, AMG900 demonstrates potent inhibitory efficiency in paclitaxel-resistant tumor cell lines and xenografts [185]. A clinical trial in patients with recurrent ovarian cancer has shown that combined treatment with 40 mg of oral alisertib twice daily plus 60 mg/m^2^ paclitaxel weekly shows promising antitumor activity with an increased but generally manageable safety profile [186]. Gemcitabine has also been considered for combined treatment with AKIs. In patients with solid tumors, AS703569 in combination with the standard dose of gemcitabine produces preliminary signs of efficacy [106]. In AML, alisertib increases the efficacy of cytarabine in a FOXO-dependent manner [187]. Another two clinical trials have demonstrated that alisertib plus induction chemotherapy with cytarabine and idarubicin is effective and safe in patients with AML [188, 189].

In addition, MLN8237 has a synergistic effect in association with vincristine and rituximab in aggressive B-cell NHL [190]. Researchers have applied this strategy in clinical trials. A combination of 50 mg of alisertib b.i.d. plus 40 mg of rituximab or alisertib b.i.d. plus rituximab and vincristine is well tolerated and demonstrates activity against non-germinal center B-cell DLBC [191]. In a study on Myc-overexpressing lymphoma xenografts, a combination of cyclophosphamide and MLN8237 induced complete tumor regression in all mice, leading to improvements in survival [192].

The combination of alisertib and carboplatin is selectively effective in glioblastoma patients with high tumor *O*^6^-methylguanine DNA methyltransferase expression who are resistant to standard therapy [193]. Eribulin, a microtubule-targeting drug, is used in metastatic breast cancer patients in the clinic. Combined treatment with MLN8237 and eribulin leads to a synergistic increase in apoptosis in mammary tumors as well as cytotoxic autophagy in metastases through the LC3B/p62 axis and Akt pathway [194]. In multiple myeloma, studies on combined treatment with AT9283 and lenalidomide have shown significant synergistic antitumor effects of this regimen both in vitro and in vivo [195]. Recently, two clinical trials have revealed that 60 mg/m^2^ alisertib per dose for 7 days is tolerable with a standard irinotecan and temozolomide backbone and shows antitumor activity, particularly in neuroblastoma patients with MYCN-nonamplified tumors [196, 197].

In addition to clinical drugs, AURKA inhibitors also show synergistic effects when used in combination with radiotherapy. PHA680632 treatment prior to radiation treatment leads to an additive effect in cancer cells, especially in p53-deficient cells in vitro or in vivo [198]. Another AURKA inhibitor, MLN8054, sensitizes androgen-insensitive prostate cancer to radiation; this sensitization is associated with sustained DNA double-strand breaks [199]. Two other AURKA inhibitors, MLN8237 and ENMD-2076, also enhance radiation sensitivity in cancer cells [200, 201]. A phase I trial on alisertib with fractionated stereotactic reirradiation therapy for patients with recurrent high-grade glioma has been conducted and has revealed that 40 mg of alisertib twice daily in combination with irradiation is safe and well tolerated [202]. Further exploration in the phase II trial may provide a better sense of clinical outcomes moving forward.

##### Combination of AKIs with targeted therapies

Cancer is a multistep disease involving multiple genes, so targeting multiple oncogenes simultaneously may enhance the efficiency of AKIs. HDAC inhibitors have been shown to repress the expression of AURKA in various cancer cells, and AKIs can decrease the activity of HDAC proteins, suggesting that synergetic effects could be obtained by combining AKIs and HDAC inhibitors [203, 204]. Studies have shown that the HDAC inhibitor vorinostat synergistically potentiates MK-0457 lethality in leukemia cells and breast cancer cells [205–207]. In addition, vorinostat and MK-0457 or MK-5108 combination treatment enhances lymphoma cell killing with reductions in c-Myc, hTERT, and microRNA levels [208]. A study in T-cell lymphoma has suggested that the effects of alisertib in combination with the HDAC inhibitor romidepsin are highly synergistic through modulation of cytokinesis [209]. Combination treatment with vorinostat and AMG900 produces synergistic antiproliferative effects both in vitro and in vivo [210]. A phase I study on alisertib in combination with vorinostat in patients with relapsed/refractory lymphoid malignancies has shown encouraging clinical activity with a manageable safety profile [211].

EGFR inhibitors have been a major breakthrough for NSCLC treatment. However, resistance to EGFR inhibitors through multiple mechanisms has been identified, including activation of other oncogenic proteins. One recent study has demonstrated that EGFR-mutant LUAD cells that demonstrate acquired resistance to third-generation EGFR inhibitors are sensitive to AKIs [212]. Furthermore, combination treatment with AKIs and EGFR inhibitors has been found to robustly decrease tumor growth in an EGFR-mutant LUAD PDX model [212].

Both BRD4 and AURKA are regulators of the MYC gene at the translational and posttranslational levels, respectively, and targeting both of them simultaneously may produce synergistic antitumor effects. JQ1 treatment to repress BRD4 activity together with MLN8237 treatment synergistically promotes cell death in c-Myc expressing human glioblastoma cells [213]. Combined treatment with another BRD4 inhibitor, I-BET151, and alisertib is efficacious in exerting antitumor effects against neuroblastoma with or without MYCN amplification both in vitro and in vivo [214].

To investigate whether combined treatment with a p53-activating MDM2 antagonist and senescence-inducing AKIs can be useful for melanoma therapy, two studies have been performed. One study showed that AURKA and MDM2 antagonism with MLN8237 and Nutlin-3 halted melanoma growth by inducing growth arrest and senescence, limiting the lifespans of senescent cells, and enhancing tumor immune infiltration and clearance [215]. The other study showed that combined MK-0457 and Nutlin-3 treatment activated p53-dependent postmitotic checkpoints at pseudo-G1 phase and induced proapoptotic p53 signaling and mitochondrial apoptosis in AML [216]. Other molecules, such as SRC [217], CHEK1 [218], mTOR [219, 220], WEE1 [221], PDK1 [222, 223], and MEK [224], have also been chosen as targets together with AURKA in preclinical studies.

##### Combination of AKIs with immunotherapy

Immunotherapy and specific monoclonal antibodies targeting multiple molecules have been widely used for cancer therapy. Combining AKIs and these agonists may enhance therapeutic efficacy. In human neuroblastoma cells, MK-5108 increases the efficacy of an anti-ganglioside (GD2) 14G2a antibody, which is related to a reduction in N-Myc expression and an increase in PHLDA1 and p53 protein levels [225]. In addition, combined treatment with an anti-GD2 14G2a antibody and MK-5108 leads to enhancement of the autophagy process in IMR-32 neuroblastoma cells [226]. A death receptor 5 agonist antibody has been found to initiate significant apoptosis in tumor cells undergoing therapy-induced senescence induced by MLN8237 treatment [227]. In that study, the combination group achieved remarkable tumor growth inhibition in melanoma xenografts derived from cell lines and patient tissues [227]. Another study has indicated that alisertib facilitates an anticancer immune microenvironment with decreased numbers of myeloid-derived suppressor cells and increased numbers of active CD8^+^ and CD4^+^ T lymphocytes [228]. More importantly, combined administration of alisertib and a PD-L1 antibody has demonstrated synergistic efficacy for the treatment of breast cancer cell 4 T1 xenografts [228]. Combining AKI treatment with anti-PD-1/PD-L1 immune checkpoint therapy may be a promising strategy for cancer treatment.

## Conclusions and outlooks

Activation of AURKA is responsible for the resistance of lung cancer to third-generation EGFR inhibitors [212]. Resistance initiated by AURKA may lead to tumor heterogeneity and promote the generation of distinct clones harboring different driving forces of drug resistance. AURKA attenuates the efficacy of inhibition of the PI3K-AKT-mTOR pathway, a downstream pathway of EGFR, in breast cancer [229]. These findings indicate that AKIs should be used together with oncogenic pathway inhibitors to treat drug resistance incrementally.

**Fig. 4:**
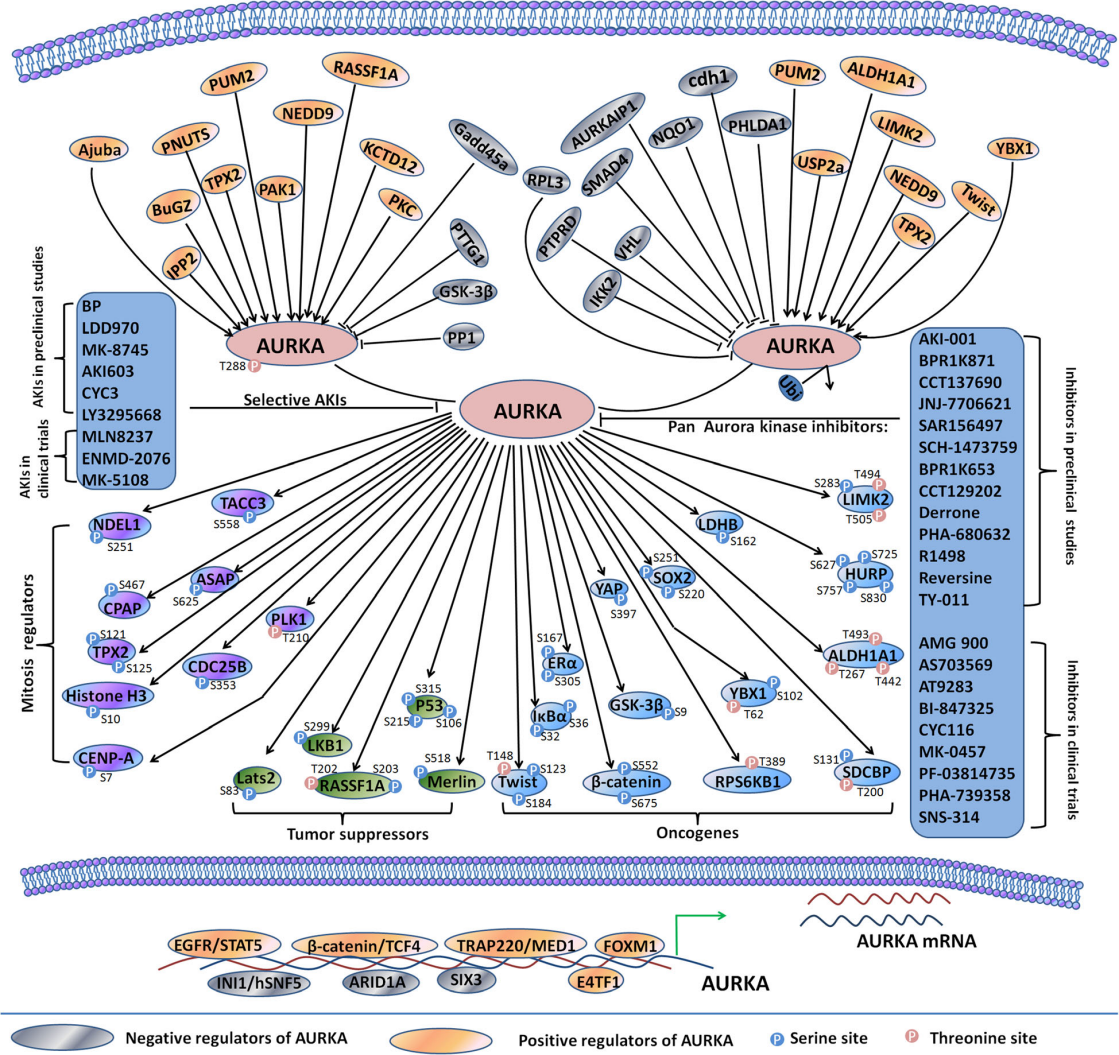
An overview of AURKA-interacting proteins and AKIs. AURKA expression is regulated at transcriptional or post-transcriptional levels and AURKA activity is tightly controlled by numerous molecules. Once activated, AURKA interacts with and phosphorylates a wide variety of proteins serving as mitotic regulators, oncogenes or tumor suppressors. Selective AKIs and pan Aurora kinases inhibitors are developed and studied in preclinical or clinical evaluation

To obtain the desired clinical benefits of AKIs, it is essential to know the pathways and proteins involved in AURKA-mediated oncogenic function. In this review, we have summarized the interactome proteins regulating AURKA or regulated by AURKA and the inhibitors targeting AURKA **(**Fig. 4**)**. Preclinical studies have shown that AKIs affect the regulation of various cellular processes, such as proliferation, invasion, metastasis, autophagy, EMT, chemoresistance and radioresistance. Furthermore, preclinical animal studies and clinical studies have illustrated the efficacy of AKIs and AKIs in combination with other standard chemotherapeutic drugs, such as paclitaxel, cisplatin and other targeted therapies.

The high toxicity of AKIs should be considered given the crucial physiological function of AURKA in normal cells. Toxicities of AKIs mainly include reversible neutropenia together with mucositis and somnolence, among which neutropenia is the dose-limiting toxicity. The predominant toxicities of AKIs reflect the mechanism of action of AURKA in highly proliferating cells such as bone marrow cells and epithelial cells. The off-target adverse events in central nervous system including somnolence and dizziness reflect the binding of AKIs to the alpha-1 subunit of the GABA-A receptor [230]. Researchers can attempt to reduce the side effects of AKIs by combining low dose of AKIs with chemotherapeutics, targeted therapies or immunotherapy. To weaken the bone marrow suppression induced by AKIs, granulocyte colony-stimulating factor (G-CSF) is administrated in conjunction with PHA-739358. In this phase I study, escalating the PHA-739358 dose until 1000 mg/m^2^ do not cause any bone marrow related toxicities, particularly neutropenia [128]. Furthermore, development of nanoparticle therapeutic carriers that are passively targeted to tumors through the enhanced permeability and retention effect may be helpful [231]. This drug delivery technology has been applied to MLN8237 and the polysaccharide nanovesicle efficiently delivers low concentrations of MLN8237 to inhibit AURKA and disrupt the anchorage-independent growth of MCF-7 cell than free MLN8237 [232].

Several methods may be taken into consideration to overcome the side effects when developing new AKIs. Researchers can take advantage of the high-resolution 3D protein structures and computer docking tools to find natural compound or FDA approved drugs that target AURKA. For example, derrone, extracted from erythrina orientalis, is screened from 100 natural substances to inhibit AURKA kinase activity and cell growth [91]. Another case is bioactive tanshinone I which is from traditional Chinese herbal medicine Salvia miltiorrhiza. Although there is no direct evidence that tanshinone I can directly target AURKA, it exhibits potent effects on growth inhibition of colon cancer [233], lung cancer [234] and breast cancer cells [235] through downregulating AURKA expression. Another way is to attempt to develop inhibitors that disrupt the interaction between AURKA and its activators. AURKA can be activated by its protein partners, among which TPX2 is the best established one. Withanone is an herbal ligand isolated from ashwagandha. Withanone is reported to bind to the TPX2/AURKA complex which results in the dissociation of TPX2 from AURKA and disruption of mitotic spindle apparatus in cancer cells [236]. Furthermore, due to the fact that AURKA exerts its function through specific substrates in certain cancers, inhibition of AURKA substrates instead of targeting AURKA kinase activity may decrease the adverse effects.

The tumor types that most likely respond to AKIs should also be studied in order to obtain the desired clinical benefits. In one preclinical study, 29 breast cancer cell lines are evaluated for the sensitivity to AURKA inhibitor ENMD-2076 [157]. ENMD-2076 shows stronger activity in cell lines lacking estrogen receptor expression and HER2 expression [157]. Furthermore, in the triple-negative breast cancer cells, cell lines with a p53 mutation and increased p53 expression are more sensitive to ENMD-2076 than cell lines with decreased p53 expression [157]. Further studies are required to establish specific biomarkers predicting whether patients will respond well to AKIs.

## Data Availability

Not applicable.

## Abbreviations

AURKA: Aurora kinase A
AKIs: AURKA inhibitors
EMT: Epithelial-mesenchymal transition
TCGA: The Cancer Genome Atlas
ACC: Adrenocortical carcinoma
BLCA: Bladder urothelial carcinoma
BRCA: Breast invasive carcinoma
CESC: Cervical squamous cell carcinoma
CHOL: Cholangiocarcinoma
COAD: Colon adenocarcinoma
DLBC: Diffuse large B-cell lymphoma
ESCA: Esophageal carcinoma
GBM: Glioblastoma multiforme
HNSC: Head and neck squamous cell carcinoma
KICH: Kidney chromophobe carcinoma
KIRC: Kidney renal clear cell carcinoma
KIRP: Kidney renal papillary cell carcinoma
LGG: Brain lower grade glioma
OV: Ovarian serous cystadenocarcinoma
MESO: Mesothelioma
LIHC: Liver hepatocellular carcinoma
LUAD: Lung adenocarcinoma
LUSC: Lung squamous cell carcinoma
PAAD: Pancreatic adenocarcinoma
PRAD: Prostate adenocarcinoma
PCPG: Pheochromocytoma and paraganglioma
READ: Rectum adenocarcinoma
SARC: Sarcoma
SKCM: Skin cutaneous melanoma
LAML: Acute myeloid leukemia
TGCT: Testicular germ cell tumors
THCA: Thyroid carcinoma
THYM: Thymoma
STAD: Stomach adenocarcinoma
UCEC: Uterine corpus endometrial carcinoma
UCS: Uterine carcinosarcoma
UVM: Uveal melanoma
RT: Rhabdoid tumor
Az1: Antizyme1
APC/C: Anaphase-promoting complex/cyclosome
NSCLC: Non-small-cell lung cancer
KEGG: Kyoto Encyclopedia of Genes and Genomes
GO: Gene Ontology
5-FU: 5-fluorouracil
IC50: Half-maximal inhibitory concentration
NHL: Non-hodgkin lymphoma
CML: Chronic myeloid leukemia
PDX: Patient-derived xenograft
PFS: Progression-free survival
PTCL: Peripheral T-cell NHL
RECIST: Response Evaluation Criteria in Solid Tumors
AML: Acute myeloid leukemia
EOC: Epithelial ovarian cancer
MTD: Maximum tolerated dose
